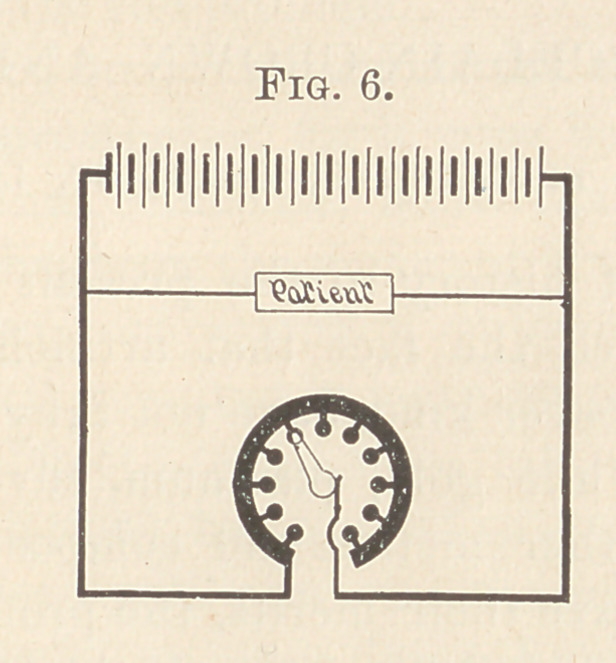# Methods of Controlling the Electric Current in Cataphoresis

**Published:** 1897-05

**Authors:** William St. George Elliott


### METHODS OF CONTROLLING THE ELECTRIC CURRENT IN CATAPHORESIS.

BY WILLIAM ST. GEORGE ELLIOTT, JR.

In this paper it is proposed to go into a comparative study of the principles employed in controlling the electric current in *cataphoresis as applied to dentistry.*

In general, it may be said that all electricity derived from batteries or constant-current dynamos is identical, as far as we need concern ourselves, and that the same laws apply to both. The strength of the dynamo or street current, its liability to a sudden and possibly dangerous increase of potential, as well as possible variations within small limits, render it necessary to consider both separately.

Electrically, any principle controlling the current as well as another principle is equal to it in efficiency. Practically, however, there are vast differences in apparatus, even when working on the same principle. Mechanically, that apparatus is the most perfect which thoroughly fulfils the requirements, while having itself the simplest and fewest parts.

The requirements of a perfect dental apparatus are:

The apparatus should be capable of turning on or shutting off the current without shock to the patient, and during the early stages of an operation should permit of very gradual increase of current strength. It should be capable of supplying a maximum pressure of fifty volts. The movement of one handle should be all that is required to control the whole current. And last, though of great importance, and usually overlooked by the manufacturers, the apparatus should occupy as little cubic space as possible.

In very sensitive cases—that is, in cases not necessarily very sensitive to the bur, yet very sensitive to the current—it has been found that a current of one-thirtieth milliampère, if suddenly applied, causes a slight shock. This makes it necessary that when we apply the electrodes the patient should not be subject to over one-tenth volt pressure to begin with. The average resistance of a tooth is probably over thirty thousand ohms. In some cases, however, where the cavity is a deep one and the apical foramen large the resistance may not be over three thousand ohms. These cases must, of course, be provided for in a good apparatus.

The current is apt to be painful in the early stages of an operation, and less so near the completion of anæsthesia. It is thought that the reason for this is that the current in passing from the electrode through the body must first traverse only a few cells, and that these increase in number and consequent area the farther it goes into the body. Now, it is a well-known fact that a cell can only stand a certain strength of current without pain, and that the amount depends on the kind and condition of the cell. Consequently, if the current must first pass through a few cells, and these are sensitive to the current, only a small amount can painlessly pass. As the cataphoric effect penetrates, however, it deprives the cells of sensation, thus enabling more current to be passed. This is accomplished by creating a greater difference of potential at the ends of the cells, which, of course, induces the current to spread and seek additional paths, and so on till the full current is passing.

The current for our purpose is controlled by resistances placed in various ways in the circuit. These resistances are usually capable of being switched in or out of use. The combined resistances and switch controlling the same is called a *rheostat*.

Fig. 1 shows diagrammatically the electrical connections for any form of rheostat.

Fig. 1.

The current follows the path indicated by the arrows. *A* is a body of high resistance. The steps *B* are connected to *A*. *E* is a rotable arm collecting the current from the steps with which it is in contact. *F* is a brush collecting the current from the arm. When the arm is at *H* all the resistance is in use, and the smallest possible current is passing; when at *F* all the resistance is out and the largest possible current is passing.

The resistant part of rheostats is either made of a homogeneous body of high resistance, such as carbon, on which the arm slides, called here *sliding rheostats*, or the body is made of a number of high resistances connected together, the arm connecting with two or more at a time, called *step rheostats.*

Sliding rheostats, when properly made, control the current by amounts corresponding with the movement of the arm. Theoretically, it is possible to vary the current to any degree of fineness by moving the arm ever so little. Practically, this form is often made of carbon of unequal thickness and uneven shape, thus causing the current to vary by jumps. Another objection is that the arm slides on the carbon, thus coating it with a thin metallic film, which, of course, alters the resistance.

Step rheostats are made with metal-wearing surfaces, and for this reason are far more reliable than the sliding form. They are, however, open to the objection that unless the resistance between the steps is small, the sudden variation is apt to be painful. This makes it necessary to have a large number of steps, and this is only possible in a few of the more compact rheostats on the market. The wide range possible, however, as well as great reliability makes this form generally preferred. But those forms in which the sliding contacts are boxed up so that no one but the makers can inspect the interior arrangements cannot be left too severely alone. Human handicraft is fallible, and therefore no matter how carefully such devices may be said to be made, accidents will happen, and when they do, however trifling the damage, the apparatus must go to the makers for repairs.

All rheostats for dental purposes should be made to turn on the current by very small steps when a small current is passing, and by progressively larger steps as more current is turned on. The first few early steps should vary by one-tenth volt, and the last step by say two volts, when fifty volts is the maximum.

Most rheostats alter the voltage of the steps by a regular and fixed amount. A far better way is to alter the voltage by a regular percentage. Thus, if we should begin with 0.100 volt, the next step would be, say, 0.104, and the last step two volts. This is supposing we have a maximum of fifty volts, and at all times take four per cent. of the current as the amount of increase for the next step.

There are two general methods employed in regulating the current. In one all the current passes directly through the patient,—the *direct method.* The other divides the current and passes the current traversing one of the branches through the patient, the other branch being used, by means of a rheostat, to regulate the amount of current passing through the first branch,—the *shunt method.*

DIRECT SYSTEM,—USING A BATTERY.

Fig. 2 shows the general arrangement for such a system. As will be seen, the current passes directly through the patient as well as the rheostat. In any closed circuit the current passing any point at any time is equal to that passing any other point at

Fig. 2.

*A* represents a battery of cells connected together in sections, and in lots of four each. *B* is the cell-selector. The arms *C*, *D* are insulated from each other. By moving the arms it is possible to select any lot of cells, or any number of lots. The cell-selector roughly controls the current strength; the rheostat *E* controls the current within the limits desired.

the same time. Now, if we wish to increase the current we must increase the pressure or diminish the resistance. There is no other way of doing it. Again, the strength of current passing through any particle depends on the difference of pressure at the points of entry and exit. If we wish to double the current we must double this difference. Conversely, if we double the current we have doubled the difference, or it would not force twice the current through. This is mentioned to show that the so-called volt-selectors have no advantage in this respect over the direct controllers. A numerical example may perhaps show more clearly how impossible it is to alter the current passing through the patient without altering at the same time the difference of pressure to which the patient is subject. In the examples that will be given it was thought best to neglect the internal resistance of the battery as well as the resistance of the line. In doing this we will not affect the general correctness of the conclusions, while not to do it would unnecessarily complicate matters. We will also take it for granted that the resistance of the patient remains constant.

*Example.*—Let us suppose we have a battery with sufficient cells in use to give a pressure of 10 volts, and have in circuit a rheostat of maximum resistance of 100,000 ohms, and a patient of 10,000 ohms resistance. The current passing through the patient will be

($C = \frac{E}{R} = \frac{10}{110000} = \frac{1}{11000}$) $\frac{1}{11}$ of a milliampère, and the patient will be subject to a pressure of ($E = CR = \frac{10000}{11000} = \frac{10}{11}$) practically one volt as soon as the electrode touches him. Now, let us take out all of the 100,000 resistance of rheostat; the patient will then receive a current ($C = \frac{E}{R} = \frac{10}{10000} = \frac{1}{1000}$) of one milliampère and a pressure of ($E = CR = \frac{10000}{1000} = 10$) ten volts. If we used fifty volts in the above example we would get a current of one-half a milliampère and a pressure of four and one-half volts as soon as the electrodes touch the patient, even with all the resistance of the rheostat in use.

There are few rheostats on the market suitable for dental use having a maximum resistance of over one hundred thousand ohms. The above examples show that with only ten volts the patient would receive one volt pressure as soon as the electrode touched the tooth. This is ten times too much for some cases, so that with such a patient and rheostat we could only use one or two cells to begin with. It is for this reason that a cell-selector is necessary.

DIRECT SYSTEM,—USING THE STREET CURRENT.

The usual pressure for incandescent circuits is one hundred and ten volts; fifty volts, however, is sufficient for all dental use, including bleaching. We therefore have sixty volts more pressure than we need, and this in this system can only be got rid of by placing resistances in the circuit so as to choke down the pressure just that much. In considering this system applied to batteries we saw that in the early stages even ten volts would cause a shock. Consequently, it is as necessary with the street current as with batteries to roughly control the voltage before it reaches the fine rheostat. This is done by placing a second rheostat in circuit.

Fig. 3 shows such an arrangement.

Let us now consider what resistance the circuit must give to the current. If we wish to send a minimum current of $\frac{1}{30}$ milliampère, the total resistance, including patient, must be ($R = \frac{E}{C} = \frac{110}{\frac{1}{30000}} = 3{,}300{,}000$) three million three hundred thousand ohms. This resistance is very large.

If we are sending a certain current through the patient, and any variation of potential should occur in the main line, the increased pressure must increase the current going through the patient by an amount exactly proportional to this variation. Consequently, if the pressure in the line be doubled, the patient would receive double the current he did before. If the wire should come in contact with a trolley wire the increase would be four hundred and fifty per cent., provided the fuses in the main line did not burn out. Practically, however, these fuses do burn out and turn off the current when only a slight increase occurs in the pressure. Indeed, the modern magnetic cut-offs can be adjusted to break the circuit at any desired increase of pressure

Fig. 3.

The fusible plug is adjusted to fuse if more than the previously determined current passes. *C* is a rheostat adapted to roughly control the current, by jumps of, say, ten volts at a time. *D* controls the current uncontrolled by *C*.

above the normal. These cut-offs are placed on every properly equipped line. We see from the above that there is practically no danger in using the street current as far as variations in the line are concerned. The variations within small limits may be so frequent, however, that they destroy the utility of the apparatus. The cataphoric lines should have fuses adjusted to burn out at from five to ten milliampères, and should also be provided with a lightning arrester. The lightning might jump the fuse posts, but with the modern magnetic arresters there is little danger of this. There is, however, always a certain element of danger from lightning during a thunder storm if the lines are not underground. When underground there is absolutely no danger from this source.

### SHUNT METHOD,—WITH BATTERY.

Fig. 4 shows such a system in outline. As will be seen, the current is split into two branches, one going through the patient and the other through all the resistances of the rheostat.

When the arm is at one terminal, *A*, there is, obviously, no electrical resistance between it and the terminal. When it is away from the terminal there will be a certain electrical resistance between the two, and this will increase the farther the arm is from the terminal. Now, we have seen that a resistance needs pressure to overcome it, and as the main current is flowing through the rheostat, it is evident that the farther the arm goes from *A* the greater the difference of pressure between it and the terminal. But the arm and the terminal form the respective terminals of the shunt or patient's line, and therefore the patient is subject to an increased difference of pressure the farther the arm goes from the

FIG. 4.

*B* represents a battery of cells; *S*, a switch; *C*, a rheostat in which the main current passes through all the resistance. The shunt branch is connected to the arm, and one terminal, *E*. The unequal thickness of the resistant material represents diagrammatically its varying resistance.

terminal *A*. It is on this old and well-known electrical principle that the so-called volt-selectors work. The voltage is 0 when the arm touches the terminal *A*, and depends for its maximum strength on the strength of the battery. If the battery is strong enough to supply sufficient current for both branches the maximum potential of the terminals of the shunt circuit is independent of the resistance of the rheostat. As all batteries are capable of supplying more current than is needed in cataphoresis, and as the resistance of the rheostat can be large, the potential difference of battery will practically be unchanged by the additional current sent through the patient. The maximum potential on shunt circuit will also be that of the battery terminals.

In this system, then, we divide up the whole potential, as no matter how great the potential we always get 0 at one end and the full possible pressure at the other. In this respect this system is far superior to the direct. If we have a rheostat with say 100 steps, and a pressure of five volts, each step will vary the pressure by an average of $\frac{5}{100} = \frac{1}{20}$ volt. If we have 50 volts each step will vary the pressure by $\frac{50}{100} = \frac{1}{2}$ volt on an average, but it is best to make this variation very much less in starting, and several times greater than one-half volt when all the current is flowing; though the average may still be one-half volt. This shows that unless the steps are very numerous (a minimum of 100) a cell-selector is required. The divisions of the cells, however, need not be as numerous as in the direct system. The objection some raise to this system is that a part of the current is wasted. This, however, is of little moment, as the current in the main branch need not exceed two or three milliampères. A more serious objection is the fact that a switch is needed to turn on and off the current. The dentist is apt to forget that the current is on and leave it on, only to find when next he needs the battery that the battery is much weakened if not entirely used up. But this can easily be remedied by making the arm move the switch.

SHUNT METHOD,—WITH STREET CURRENT.

This method is capable of numerous variations, one of which is shown in Fig. 5. As will be seen, the fine rheostat is in shunt with the main line. It is not always necessary to have a rheostat in the main circuit. A resistance of two hundred ohms, if placed in this

FIG. 5.

*A* and *B* represent the high- and low-tension street wires; *C*, a switch; *D*, a coarse rheostat; *E*, a shunt line with the fine rheostat in circuit. The patient is in shunt from the fine rheostat.

circuit beside a two hundred and forty ohm resistance, will do to take off fifty volts from. This allows a current of one-fourth ampère to pass through the main circuit. The patient should always be on shunt from the fine rheostat so as to be able to begin operation without shock. The potential of the shunt varies according to the current passing through the main circuit and the resistance of the circuit. The resistance is always constant, therefore the only way the pressure can be varied when the arm is not moved is by varying the current. This shows that if variations occur in the main line they will be transmitted to the shunt circuit, and through the patient, in exactly the same ratio as in the direct system, notwithstanding the claim of various firms to the contrary.

Fig. 6 shows another method. Here it will be seen that in order that the patient should receive little current, most of it must go through the rheostat. The amounts the patient and rheostat will receive will be inversely as their resistances. As in a battery it is necessary to economize in current, it is essential to put an additional resistance in the circuit to prevent the flow of too much current when the resistance of rheostat is at a minimum. This

Fig. 6.

resistance, theoretically, need only be used in starting, but as it cannot be suddenly thrown in or out without shocking the patient, it is necessary either to keep the resistance permanently in circuit or make it a second rheostat. If kept permanently in circuit it needs additional cells to overcome the resistance, and if a rheostat is used it adds unnecessary complication. This method then offers no advantage over the usual methods previously described and is neither so simple nor so efficient.

We have now seen that neither the direct nor the shunt method overcomes the fluctuations in the street current. These are large in some cities and small in others, but are never entirely absent. For this reason, as well as to eliminate the possible danger from lightning, it is well to use batteries.

The shunt method is preferable in all cases, for the reason that it divides up the whole pressure. Especially is it valuable if the street current be used, as in that case enormous resistances are not needed. Another advantage this method possesses is that the total resistance of the rheostat is not material, except in that if too small more current is wasted. As the dentist's spare space near the chair and within reach is always limited, the battery should be as small as possible. It would seem quite feasible to make a compact instrument that would regulate the current as the conditions in dentistry require, and which would control the current entirely by the movement of one arm. This arm should turn the current on and off as well as regulate the strength. With a good form of cell the voltage is fairly constant, even for years. It is therefore possible with such a battery when using the shunt method to have the arm indicate the exact voltage the patient is subject to.

## Figures and Tables

**Fig. 1. f1:**
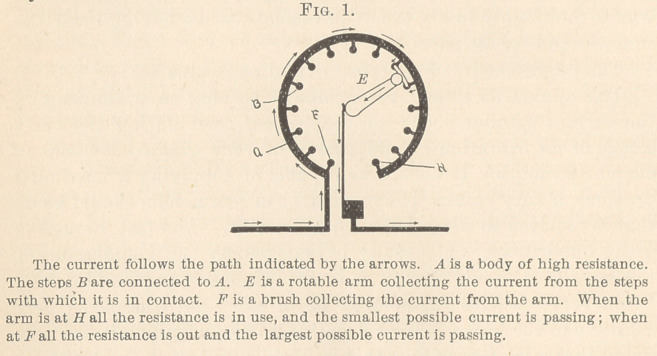


**Fig. 2. f2:**
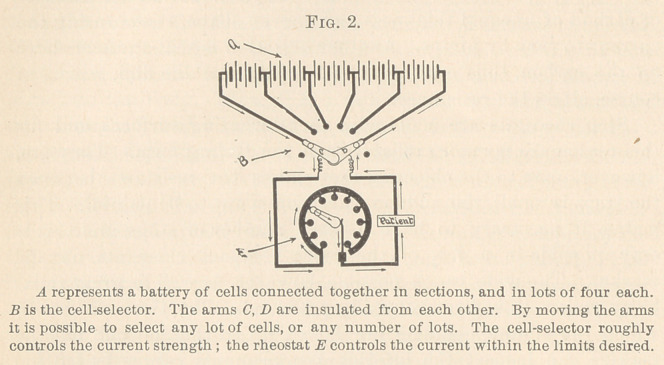


**Fig. 3. f3:**
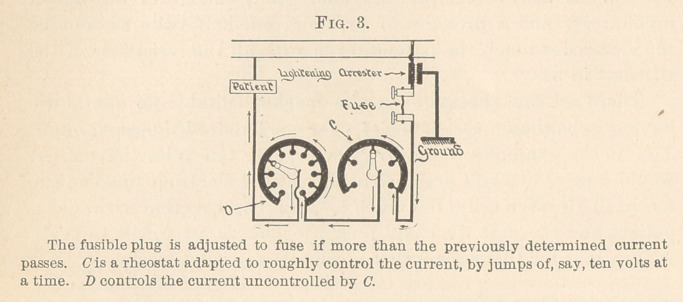


**Fig. 4. f4:**
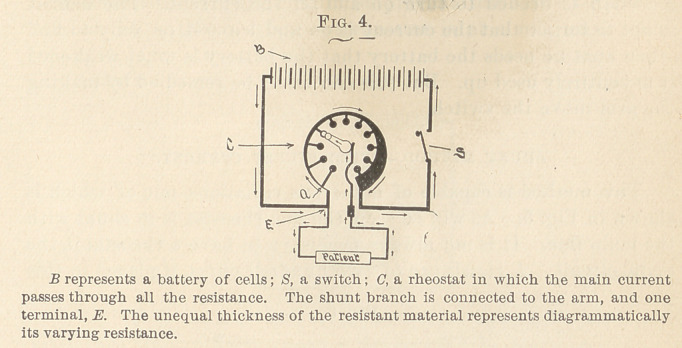


**Fig. 5. f5:**
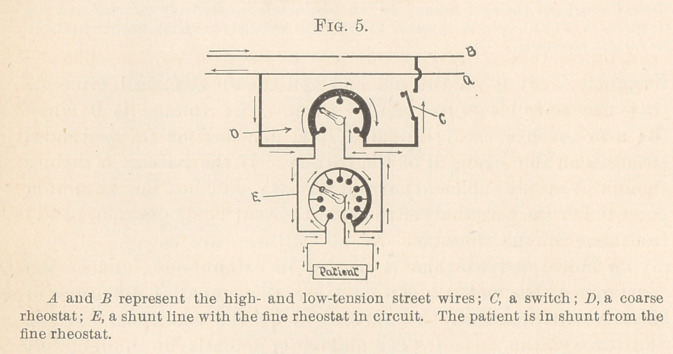


**Fig. 6. f6:**